# What we have changed our minds about: Part 2. Borderline personality disorder, epistemic trust and the developmental significance of social communication

**DOI:** 10.1186/s40479-017-0062-8

**Published:** 2017-04-11

**Authors:** Peter Fonagy, Patrick Luyten, Elizabeth Allison, Chloe Campbell

**Affiliations:** 1 Research Department of Clinical, Educational and Health Psychology, University College London, London, UK; 2 Faculty of Psychology and Educational Sciences, KU Leuven, Leuven, Belgium

**Keywords:** Borderline personality disorder, Resilience, Epistemic trust, Mentalizing, Attachment, Psychopathology

## Abstract

In Part 1 of this paper, we discussed emerging evidence suggesting that a general psychopathology or ‘p’ factor underlying the various forms of psychopathology should be conceptualized in terms of the absence of resilience, that is, the absence of positive reappraisal mechanisms when faced with adversity. These impairments in the capacity for positive reappraisal seem to provide a comprehensive explanation for the association between the p factor and comorbidity, future caseness, and the ‘hard-to-reach’ character of many patients with severe personality pathology, most notably borderline personality disorder (BPD). In this, the second part of the paper, we trace the development of the absence of resilience to disruptions in the emergence of human social communication, based on recent evolutionary and developmental psychopathology accounts. We argue that BPD and related disorders may be reconceptualized as a form of social understanding in which epistemic hypervigilance, distrust or outright epistemic freezing is an adaptive consequence of the social learning environment. Negative appraisal mechanisms become overriding, particularly in situations of attachment stress. This constitutes a shift towards a more socially oriented perspective on personality psychopathology in which the absence of psychological resilience is seen as a learned response to the transmission of social knowledge. This shift in our views has also forced us to reconsider the role of attachment in BPD. The implications for prevention and intervention of this novel approach are discussed.

## Background

Bringing together the threads of the argument we built in Part 1 of this paper, we propose that the common variance revealed by bi-factor studies of psychopathology indicates a shared variance in resisting socially expectable adversity. Moreover, persistent psychological distress associated with personality disorder (PD) has as a common element diagnostic criteria that we may particularly expect to see in BPD, making BPD features the core features linked to persistence of psychiatric problems. So far, we have outlined a model that inverses this vulnerability from one focused on the common characteristics of the pathological condition to an alternative perspective that highlights the absence of resilience as the shared cause. Following Kalisch et al.’s persuasive model of resilience [1], we argued that the persistence of psychopathology, as observed prototypically in BPD, results from a pervasive limitation on the appraisal of stressful social experience, which could be linked to limitations in the capacity to mentalize.

What may explain this absence of capacity to reappraise stressful social experiences? Here, recent evolutionary and developmental accounts of the emergence of epistemic trust in humans may provide important answers. These views also, as we will demonstrate, necessitate a shift in our perspective on the role of attachment in BPD. Put briefly, the theory of epistemic trust posits that the human infant – most usually first within the context of early attachment relationships – is instinctively inclined to develop openness to the reception of social communications from their primary caregivers. Stated otherwise, epistemic trust is an adaptation allowing the infant to receive social knowledge from their better-informed elders [2], enabling them to benefit from the complex edifice of human knowledge that their immediate culture has available to them.

There are two possible bases on which cultural knowledge can be accepted by a learner as credible: they can either work it out for themselves (which is time-consuming, difficult, and often impossible) or they can rely on the epistemic trust they have in the authority of the communicator [3, 4]. Trusting the communicator means that the learner does not have to go back to first principles each time they encounter novelty: a strange-looking tool without a self-evident purpose is accepted as being used as described by a trusted elder, because they have said so [5]. Being told in this way is enough, and saves an awful lot of time and effort, and indeed possibly allows the infant to grow up and build upon or revolutionize the use of the tool in question. This capacity to teach and learn social knowledge largely underpins the evolution of human culture [6]: it has been proposed that this form of cultural evolution, based on the transmission of knowledge via epistemically trusted communication, emerged during the late Pleistocene era [7].

The internalization of knowledge about the social world constitutes a particular kind of learning: it involves encoding the piece of knowledge as *significant*, *relevant* to the recipient and *socially generalizable* – that is, as an accepted and reusable piece of cultural currency. This specific form of learning is stimulated by ostensive cues generated by the communicator [8, 9]. Such cues trigger a pedagogic stance in the recipient, priming them to regard forthcoming communications as significant. Human infants display species-specific sensitivity and deference to non-verbal ostensive cues, such as eye contact, turn-taking contingent reactivity, being called by their name, and the use of a special tone of voice (‘motherese’) by the communicator [10, 11]. These ostensive cues have in common the quality that the recipient is recognized as a subjective, agentive self. Once epistemic trust is stimulated in this way, the channel for the transmission of knowledge is opened. Mimicry may be protected by human evolution because it generates epistemic trust, inevitably signalling recognition in the child by the imitating adult. A social smile (recognition of the self by the other) probably increases the tendency for imitation because the smile generates epistemic trust and opens the communication channel to receive knowledge.

It has been argued that this mechanism for opening the epistemic channel exists because it cannot be left open by default: it is adaptive for humans to adopt a position of epistemic vigilance unless they are reassured that it is safe to do otherwise [4, 5]. The notion that children are promiscuously credulous to those around them has been disproved by ample evidence suggesting the ways in which dubious social signifiers and poor past performance may render a social communicator suspect and their assertions about the world regarded with scepticism [12, 13]. Epistemic vigilance is a necessary tool to protect against misinformation, whether as a consequence of malicious intent or incompetence on the part of the communicator [4]. Therefore, although the purpose of epistemic trust is the transmission of data, its application is a highly psychological and relational process, dependent on calculations about who is trustworthy, authoritative and knowledgeable –in other words, about whose information is worthy of being encoded as relevant and culturally significant to the self.

## Epistemic mistrust and developmental psychopathology

In situations where a young learner’s early environment is heavily populated by unreliable communicators, the opening of epistemic trust becomes problematic: it may be more adaptive to remain persistently vigilant about, or even closed off to, the communication of social knowledge. In the face of an abusive and hostile caregiver, whose intentions towards the infant or child are not benign, epistemic mistrust becomes entrenched as an appropriate adaptation that has been prepared by natural selection.

Consistent with these assumptions, an accumulating body of evidence indicates that childhood maltreatment, broadly defined, can have a negative impact on several aspects of social-cognitive competencies in individuals who have not yet been explicitly diagnosed with a mental disorder [14–17]. Young maltreated children display impairments with regard to several indices of mentalizing: (a) they engage in less symbolic and less child-initiated dyadic play [18, 19]; (b) they sometimes fail to show empathy when witnessing distress in other children [20]; (c) they have poor affect regulation, which contributes to psychopathology and peer rejection in later life [21–24]; importantly, (d) they make fewer references to their internal states [25]; and (e) they struggle to understand emotional expressions, particularly facial expressions [26, 27]; this latter feature has been observed even in studies that controlled for verbal IQ [28, 29]. The impact of maltreatment reaches into adulthood. A large-scale study of 5000 adults [30] found that maltreatment by parents in childhood was strongly associated with adult variations in theory of mind, or mental-state inferencing, as well as self-reported levels of social affiliation (social motivation and social support). Interestingly, this study found that face discrimination and face memory abilities in adulthood were relatively unaffected by early adversity. The findings confirm that social cognition may be the domain that it is particularly vulnerable to the effects of adverse childhood environments.

Impairments in epistemic trust are a further, and perhaps more damaging, long-term sequel of the experience of childhood maltreatment. Epistemic hypervigilance can manifest as the overinterpretation of motives, which can take the form of hypermentalizing [31, 32], or pseudomentalizing [33]. There is significant evidence that the quality of the relationship of a child to a given communicator determines the extent to which they acquire and generalize information from that communicator [34–36]. When in a state of epistemic hypervigilance, the recipient of a communication assumes that the communicator’s intentions are other than those declared, and the information is therefore not treated as being from a deferential source. Most typically, epistemic mistrust manifests as the misattribution of intention and the assumption of malevolent motives behind another person’s actions, and therefore treating them with epistemic hypervigilance (or conversely, in some instances, excessive inappropriate epistemic trust). There is evidence to suggest that a hypermentalizing stance is more characteristic of BPD in adolescence [31, 32]. It is possible that this hypermentalizing typically subsides into a flatter profile of outright epistemic mistrust as the individual matures. We speculate that this pattern may partially account for the common life-course history of BPD symptoms, which demonstrates a reduction in impulsive symptoms over time but no lessening of the affective and social symptoms associated with BPD.

In a state of epistemic mistrust, the recipient of social communication may well understand what is being expressed to him/her, but he/she cannot encode it as relevant, internalize it, and appropriately reapply it. The consequence is that the regular process of modifying one’s stable beliefs about the world in response to social communication is closed down or disrupted. This generates the quality of rigidity and being ‘hard to reach’ that therapists have often described in their work in the field of PD [37]. Change cannot happen in the therapeutic setting because, although the patient can hear and understand the communications transmitted to them by the therapist, the information cannot be accepted as relevant to them and generalizable to other social contexts. The persistent distress and social dysfunction associated with PDs is the result of the destruction of epistemic trust in social knowledge of most kinds.

PD may therefore be best understood as a failure of communication arising from a breakdown in the capacity to forge learning relationships. We believe that this quality underlies the painful sense of isolation that characterizes the subjective experience of an individual with BPD.

## Reconsidering the role of attachment

The change of emphasis in relation to the role of attachment theory in the aetiology of PD we will consider in this section speaks to some of the long-standing criticisms of attachment theory that emerged from two directions: psychoanalysis and anthropology. The psychoanalytic criticism of attachment has tended to take the position that attachment theory is too mechanistic and reductionist; that its broad classifications leave attachment unable to engage with the subtlety and complexity of individual human subjectivity. These arguments have been well rehearsed [38, 39]. Meanwhile, anthropologists have suggested that attachment theory disallows other kinds of complexity: those that arrive from cultural differences and varying environmental imperatives. Varying contexts might indeed generate different family configurations and caregiving expectations and structure, for example, alloparenting [40]. As another example, the fluid capacity of caregivers to attach, disengage and reattach across their lives has been compellingly described by the anthropologist Scheper-Hughes in her work on mothering in an acutely impoverished milieu, where she observed mothers facing the death of their infants with apparently little sorrow, but become loving mothers to subsequent children or children who, having previously been given up on, went on to survive [41]. Similarly, historians have traced high rates of infanticide in many cultures (30–40% in early 19th century Milan, for example [42]). Indeed, early historians of childhood, such as Philippe Ariès [43] and Lawrence Stone [44], characterized it as a state of unremitting abuse and brutality. Stone argued that the high levels of infant and child mortality in the pre-industrial era precluded the investment of love and affection in children that we would now consider normative [44]. More recently, this depiction of the experiences of children in the past has been displaced by a more subtle and complex portrait of how parents have historically perceived and related to their children [45]. Ample examples have been found of the ways in which children were recognized, loved, protected and mourned for by their caregivers (e.g. [46, 47]). These academic skirmishes over the sameness and difference of being a parent and a child across time, and the co-existence of love and violence in human experience, should not surprise us from a clinical point of view: they are in keeping with our understanding of attachment as a universal human (and indeed mammalian) instinct, while still allowing us to recognize, for example, the high rates of infanticide that historians have traced in some periods [42]. In all but the most cases extreme childrearing scenarios, attachments of some style do form; but it is possible that different social environments are likely to trigger different attachment styles as being more adaptive to each environment.

The attachment style to which the child is exposed may be protective of the child, even if it is harsh or cruel. We thus suggest that attachment styles are themselves one piece of social communication that the familial context is promoting about the most effective way to function in the prevailing culture. Attachment is part of a social signalling system telling the infant or young child to prioritize developing specific mentalizing capacities and particular patterns of behaviour. The family environment associated with BPD may entail triggering a particular style of adaptation to ensure survival to reproduction, albeit one that causes pain to the individual and is challenging to the immediately surrounding environment. For example, risky sexual behaviour in adolescents with a childhood history of neglect may be a way of increasing the likelihood that they will contribute to the gene pool. Such behaviours are resistant to change because the adaptation is triggered by natural selection; the individual’s genes ‘communicate’ that this is most likely to ensure survival (of the genome) [48]. Lower levels of mentalizing, greater aggressiveness and higher sensitivity to perceived threats may be adaptive responses to certain cultural environments. Natural selection has charged families with psychologically enculturating their children to maximize their likelihood of survival. Social learning from the immediate family and culture can help us account for the relationship between individual behaviours and the culture that engenders them. Low levels of interpersonal understanding, or even frank attacks on the self-awareness of individual family members, may be biologically successful, evolutionarily selected strategies. A stance of dismissing attachment and non-mentalizing is not experienced as a deficit by the person adopting this stance, but rather as the most appropriate strategy to ensure their survival. It further follows that if mentalizing interventions are to succeed with children, they need to occur in the context of the family [33] and enhance the quality of mentalizing within the family system to which children are oriented to acquire social expectations.

At a theoretical level, this change in focus involves a certain reconfiguration of the role of attachment in developmental psychopathology. Like other authors [49], we have previously placed considerable weight on the nature of attachment disorganization in our accounts of BPD based on the mentalizing model [50]. We maintain that the role of attachment is highly significant in the developmental origins of PD. However, we argue that its role might perhaps be best understood as only one (albeit very important) form of content learned from the social environment. This is congruent with recent work suggesting that the relationship between infant attachment status and later outcomes is more complicated than that suggested by early attachment studies [51]. Other findings have suggested limited evidence for linking childrearing environments to later outcomes and the fluctuating significance of infant attachment style across the life trajectory. For example, in infancy, the role of genes in determining security or insecurity of attachment is negligible and the childrearing environment is critical [52]; however, in adolescence, the impact of genetic factors rises considerably, such that they predict 38 and 35% of security and insecurity, respectively [51]. Meanwhile, parental sensitivity – previously considered key for the transmission of attachment security in infancy (see a major meta-analysis by Verhage et al. [53]) – may have other functions beyond ensuring secure attachment, although this function is, of course, an important one. The relationship between parental sensitivity and developmental outcomes, according to recent and highly compelling findings by Kok et al. [54], may be more general and structural than can be captured by infant attachment status: these findings indicate that normal variation in maternal sensitivity is related to markers of optimal brain development. This suggests that the parenting environment supports the neurobiological architecture of higher-order cognitive function upon which the capacity to mentalize depends.

We suggest that the relationship between parental sensitivity, attachment and epistemic trust lies in the way in which epistemic trust in most normal circumstances develops in the context of attachment relationships. Secure attachment, which provides mostly consistent contingent parental responses to the child, also provides mostly consistent ostensive cueing and therefore the most fertile ground in which epistemic trust can emerge and subsequently generalize to new relationships. This, of course, follows Bowlby’s description of internal working models [55]. Attachment to a safe, sufficiently reliable and mentalizing caregiver provides the child with a sense of agency that allows the child to have some confidence both in their own interpretation of the social world, and in the good faith and general accuracy of their caregivers’ communications [56].

The role of attachment in our conception of personality has shifted as we have increasingly come to regard the conceptualization of linear causation in psychopathology as unhelpful; instead, we conceptualize the perpetuation of PD being driven by loosely coupled interacting systems working in a circular way. A linear approach would posit that the capacity for mentalizing is vulnerable because of the social-emotional quality of early attachment experiences; partial, erratic mentalizing turns into an interpersonal vulnerability whereby a person feels interpersonally brittle because they cannot reliably process the psychological meaning of social experience, and vulnerable because they cannot process their own emotional reactions to these experiences.

Evidence suggests that attachment stress derails mentalizing judgments [57]; working in the other direction, attachment schemas predict mentalizing in adolescence [58, 59]. According to this model, mentalizing and emotional regulation compete, and attachment insecurity has a catalytic role in disrupting the development of optimal mentalizing capacity.

Mentalizing difficulties lead to affect dysregulation, which in turn further disrupts mentalizing. Wherever this cycle starts, mentalizing problems lead to interpersonal conflict and social difficulties, which generate intense (social) affect such as shame, which is inadequately contextualized because of the failure of social cognition. This affect further undermines the capacity to mentalize, which can then create further social challenges, generating interpersonal conflict that will inevitably lead to higher emotional arousal. The emotional arousal is poorly modulated and causes further disruptions of social cognition as part of a recursive process, the final outcome of which is an individual lacking the higher-order cognitive capacity necessary to withstand even everyday social adversity.

The likely interaction between a history of adversity that challenges epistemic trust and mentalizing failure as both a cause and a consequence of emotion dysregulation culminates in a stance where the individual with limited mentalizing capacity cannot reliably detect ostensive cueing and adopts what is perceived to be a maladaptive pattern of rigidity – that is, inability to change. What emerges is an (implicit) attitude of mistrust in the social environment [60] and an incapacity to learn from social experience or to modify one’s behaviour on the basis of social learning. In our view, these individuals are those with high ‘p’ scores whose disorders persist because of their inaccessibility to normalizing social influence. Their ‘impermeability’ to therapeutic influence comes not from the deep-seatedness of the pattern but its central manifestation of epistemic mistrust born of a dual core of a history of adversity and emotionally disrupted sensitivity to ostension. This is not a naive environmental theory promoting the quality of social interaction at the expense of biological factors: there is every reason to suspect that genetic predisposition, as well as the normal mixture of early environmental determinants, makes an individual more or less receptive to ostensive cues. The fact that therapeutic interventions have the capacity to promote sensitivity to ostensive cues in no way prejudges the balance of biological versus psychosocial influences on sensitivity to social cues. Because clinicians have historically linked non-responsiveness to therapeutic intervention to *characteristics of their patient* rather than *features of their own relationship to the patient*, the pattern of epistemic mistrust/hypervigilance was regarded as a feature of the most stable system they could identify in their patient – their personality. As ‘normal’ personality is in fact far from stable, consistent or unmalleable in relation to social situations [61, 62], perhaps disorders of personality are so called because, unlike normal personality, individuals with PDs have in common an absence of flexibility and great difficulty in adapting to changing social situations. Hence, epistemic mistrust may have its roots in part in disturbed attachment experiences, but ultimately it is a disorder of social communication or social learning. Its core is a compromised capacity for appropriately interpreting social actions in terms of mental states, which is what normally bolsters resilience, leaving the individual with dysfunctional social learning systems that are inadequate to assure adaptation in the face of ‘normal’ adversity.

Although this perspective has considerable bearing on our understanding of the subjective experience of BPD, it is also one that is consistent with a conceptualization of the human mind as having evolved to be highly social and culturally responsive. Therefore, it is a theory that is relevant to how we think about the relationship between the individual and culture, and it is of relevance to a much broader and more interdisciplinary way of thinking than our previous position was. This rather more systemic, less intrapsychic approach involves a repositioning of the role of attachment in developmental psychopathology to accommodate the imperatives of the wider social environment within which the dyadic relationship is located. The anthropologist Thomas Weisner expressed it thus:

The question that is important for many, if not most, parents and communities is not, “Is [this individual] child ‘securely attached?’” but rather, “How can I ensure that my child knows whom to trust and how to share appropriate social connections to others? How can I be sure my child is with others and situations where he or she will be safe.” Parents are concerned that the child learns culturally appropriate social behaviours that display proper social and emotional comportment and also show trust in appropriate other people. ([63], p. 263)

Our thinking has – albeit from a different direction – come to a similar conclusion.

## The role of systems

If the lack of resilience we associate with BPD is to be understood as an inability to access positive appraisal and the inhibition mechanisms owing to imbalances in mentalizing and the associated compromise of epistemic trust, this also has implications for the system inhabited by that individual. As outlined earlier, we suggest that ‘personality’ dysfunction persists through the self-perpetuating cycle of social dysfunction and mentalizing difficulties. The resulting heightened affect disrupts the interpersonal environment, creating social challenges that derail mentalizing and in turn undermine social functioning.

**Fig. 1:**
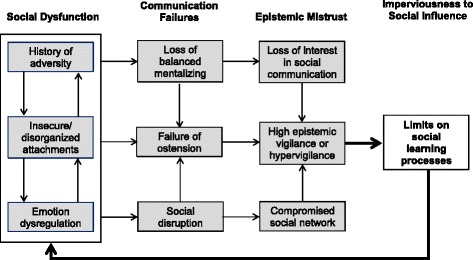
The Natural Pedagogy Model of Personality Disorder. Illustrates the interactions between social dysfunction, failure in social communication, epistemic mistrust, and imperviousness to social influence that underpin personality disorder. Emotion dysregulation, disrupted attachment histories and the disorganized insecure attachment system generate social/interpersonal dysfunction. This undermines accurate social communication, causing social disruption, the misinterpretation of social signals, and difficulty in recognising ostensive cues from others. These difficulties in the area of social communication can give rise to epistemic mistrust in relation to the social environment. This is not inherently a maladaptive process: epistemic vigilance has a natural function. However, the absence of epistemic trust sets a limit upon social learning. This can render the individual potentially unable to function effectively within their social environment and can lead to further disruption in the social network, leaving the individual increasingly isolated and prone to further social/interpersonal dysfunction

A graphical display may help to illustrate these complex interactions (see Fig. 1). Emotion dysregulation, disrupted attachment histories and the disorganized insecure attachment system interact to generate social/interpersonal dysfunction, a shared characteristic of PDs [64, 65]. Such dysfunctions are best understood as communication failures rather than as properties or characteristics of the individual suffering from PD.

The failure of communication occurs at a number of levels. First, the social disruption associated with interpersonal conflict will itself compromise the processes of social learning and, in particular, of salutogenesis (the capacity to learn and benefit from the (social) environment). This is a systemic failure of communication that may characterize a family, the members of a social group such as a gang, a social subculture, or indeed an entire culture. We will discuss such systemic failures in more detail below in terms of their impact on the network of social influence within which all socialization occurs.

Second, the loss of balanced mentalizing triggered by interpersonal conflict generally lessens interest in the content of communication and social information exchange. There is a pervasive loss of interest in intentionality; observable outcomes are gradually prioritized as indicators of attitudes and the general tenor of verbal communication is perceived as meaningless ‘psychobabble’ with few or no substantive implications for the life of the individual.

Third, social dysfunction, as well as the misinterpretation of social signals associated with the loss of mentalizing, leads to a probable failure to appropriately identify ostension – the sense that a communication is of personal relevance.

These factors (and probably many others) contribute to the individual’s failure to develop epistemic trust in personally relevant communications. Again, we are keen to point out that this is not inherently a maladaptive process. The failure to develop epistemic trust leaves the natural function of epistemic vigilance in place. It is in fact an efficient adaptation and an indication that the individual is exercising appropriate caution in relation to social influence, which we see as manifesting in the undesirable persistence of antisocial expectations or schemata and the individual’s relative imperviousness to social influence.

However, the absence of epistemic trust sets a limit upon social learning. This can render the individual increasingly ill-suited to function effectively within their social environment. Disruption of the social network within which the individual could (or perhaps should) function leaves them increasingly isolated and prone to further social/interpersonal dysfunction.

There are many levels at which systemic thinking applies to how we respond to PD. In terms of clinical work, a mentalizing team around the therapist is, we argue, essential for maintaining good practice. In the context of the persistent distress associated with PD, clinical encounters happen, by necessity, against the background of constant exposure to psychic equivalence and pretend or teleological modes [33, 66]. We suggest that it is the impact of non-mentalizing on the system of social communication, and not the unchangeability of non-mentalizing per se, that makes PDs clinically challenging conditions. One of the defining characteristics of PD is that the patterns of social dysfunction shown by the patient are enduring. Indeed, as mentioned above, BPD in particular has traditionally been regarded as an almost untreatable condition; this is one of the factors that have contributed to the stigma experienced by those receiving a PD diagnosis. However, effective therapies for BPD now exist: at least nine forms of treatment have been tested in at least 20 randomized controlled trials [67], and patients with BPD should no longer be regarded as ‘unhelpable’. We would argue that the apparent inconsistency that a condition has long been believed to be untreatable, yet appears to be more responsive to therapy than most mental disorders, is to be found to lie in the way the non-mentalizing actions of BPD patients can create non-mentalizing social systems that sustain their condition – including in the consulting room. We suggest that it is unrealistic to expect a clinician working with such patients to themself maintain an effective mentalizing stance in the medium to long term if they are not supported adequately to maintain their capacity to mentalize, ideally by a surrounding team that is not directly exposed to (and is thus protected from) the patient’s dysfunctional social system.

Systemic interventions may be required to address these problems [68]. In principle, the patient and therapist are isolated in a room, albeit with bidirectional social influence – the therapist is, after all, in a position to enhance the patient’s capacity to reflect, to question and to focus simultaneously on both other and self, inside and outside. But the reality is that the therapist becomes embedded within the patient’s social survival mechanism, which subsumes the obliteration of balanced mentalizing (normally erring on the side of being unreflective, externally focused, emotional and dominated by resonance rather than reflectiveness). The clinician’s mentalizing, even if exceptional, is unlikely to be sufficient to be able to deal with such highly intense emotional situations and conflicts. Therapists require their own system of support relationships, primarily from other clinicians, in order to scaffold their capacity to mentalize and facilitate epistemic trust.

The self-perpetuating cycle of sustained dysfunction associated with BPD and a non-mentalizing social system reminds us of the international variability in the prevalence of BPD. It has been observed that BPD is less common in non-Western societies, possibly as a result of the fact that the lack of social capital and community support characteristic of many modern or modernizing societies leaves the individual more vulnerable to impulsivity and affective instability [69]. Available prevalence data suggest that Western countries with higher levels of inequality of wealth experience higher rates of BPD [70]. The anomie of modern life – that is, a lack of social connectiveness leading to dysregulation –described by Durkheim [71], and connected by other authors with the conditions that might account for national variations in BPD [69], can be read as a description of a systemic collapse of epistemic trust. This emphasis on the role of the social environment points to the value of thinking about ways in which a social climate can be encouraged to become more mentalizing to support a change process. Families are one obvious example of a systemic arena for the promotion of mentalizing that reinforces the learning of epistemic trust. Bateman and colleagues’ development of the Families and Carers Training and Support programme (FACTS) for those supporting a family member with BPD is one example of a mentalizing intervention for the family [72].

The school is another system that seems ideal as the site for mentalizing interventions. Tellingly, evidence suggests that, of the many interventions that now exist to deal with bullying in schools, the most effective share the characteristic of involving the whole school [73]. A mentalizing-based approach, known as Creating a Peaceful School Environment (CAPSLE), is one of three bullying prevention strategies found by a large meta-analysis to be most effective [74] (the other two programmes were the Olweus Bully Prevention Program, whose generalizability has recently been questioned by Bradshaw [75], and Finland’s KiVa national anti-bullying program [76]). The mentalizing approach of CAPSLE is a systemic one, which seeks to create a mentalizing climate and a group dynamic that can resist and limit the potency and currency carried by the individual acts of violence or aggression that are inevitable in a school [77–80].

AMBIT (adaptive mentalization-based integrative therapy) is a third example of a clinical approach that combines mentalizing with thinking about the systems that surround an individual [81, 82]. Originally developed for ‘hard-to-reach’ adolescents with complex needs, AMBIT is now being applied to younger and older client groups. Such clients present with multifaceted difficulties and so tend to attract complicated multi-agency and multi-professional networks aiming to provide help. At the same time, these clients tend to be highly alienated from conventional social networks, while often forming personal relationships that carry further risks. AMBIT seeks to counter these difficulties by using a main keyworker to, where possible, simplify the individual’s experience of the complex network that surrounds them. The keyworker simultaneously seeks to support and encourage the non-professional social networks that surround the individual (e.g. the family, friendship groups or extra-curricular/activity-based groups), while also serving as a secure attachment base from which the individual might explore the social opportunities their environment presents. A final crucial component of this approach is its emphasis on the need for a supportive mentalizing system around the keyworker, given the anxieties and pressures involved in such therapeutic work.

The systemic mentalization-based interventions outlined above have in common their view of the individual as being temporarily separated from their social network, and of their capacity to form bonds of trust being shaky and prone to disruption. Without intervention, the person loses their epistemic safety net; the socially defined network of meanings is under threat. The interventions address the *network*, not just the individual or the therapist. In AMBIT, the links between the keyworker and the ‘dis-integrating’ (the term used in AMBIT to indicate the frequency with which the various agencies around a client may pull in opposite directions in their various attempts to work with the client) social care system around the family are an important focus. In CAPSLE, the non-mentalizing bully–victim–bystander is focused on by everyone within the whole school. FACTS aims to address the non-mentalizing within the family system. Common to each of these approaches is its capacity to ensure that epistemic trust – the meaningful transfer of information from one person to the other – is ultimately assured and protected. It is evident in CAPSLE where the disruption of epistemic function makes the intervention necessary; indeed, one of the outcome measures for this intervention is the improvement of children’s scores in standardized assessments of educational attainment [79]. In AMBIT, meaningful communication between different helping systems is resumed with the restoration of mentalizing. Similarly, in FACTS, with improved mentalizing the family can once again take up its function of social information transmission. It is in our opinion thus not mentalizing itself that is of direct benefit; it is the normal social functions that depend on mentalizing that bring the real therapeutic benefit.

Non-mentalizing social systems present a powerful cue that the individual is in an environment where social relations are not operating on the principle of shared goals, cooperation and interdependence. It is these behavioural imperatives that are, as Tomasello described, associated with our higher-order cognitive capacities [83]. When presented with cues that signify that we do not have access to collaborative social relations, we make cognitive adjustments, as evidenced by new research on Social Baseline Theory [84]. As a simple illustration, hills are judged to be less steep when one is standing next to a friend, and there is a dose–response effect: the longer the friendship, the less steep the hill appears to be [84, 85]. Coan et al. state that ‘The human brain *expects* access to relationships characterized by interdependence, shared goals, and joint attention’ ([84], p. 87). Violations of this increase stress and increase cognitive and physiological effort – ‘social relationships decrease the predicted cost of the environment’ ([84], p. 87). Social behaviour is so closely at the heart of the human evolutionary story that it is a fundamental instrument that humans use to ‘mitigate risk and diminish the level of effort needed to accomplish goals’ ([84], p. 87). In the absence of this social baseline, the environment is perceived to be more risky and costly in terms of effort. The accessibility of social support is one of the factors that humans – and other social animals – use in adjusting their appraisal bias.

Literature relating to research in non-human animals shows that the capacity of an organism to regulate its internal state according to evaluations of the external conditions (rather than through basic stimulus–response mechanisms) is fundamental to behavioural flexibility; it has been recently suggested that appraisal theory can be fruitfully brought into this thinking [86]. In particular, it has been suggested that cognitive biases arising from the interference of affective states, as well as genetic and environmental factors, can affect the appraisal of ambiguous situations, which subsequently shapes resilience to stressful events [86]. One example is Harding and colleagues’ classic finding that rats exposed to unstable housing conditions made more pessimistic evaluations of ambiguous stimuli, in a way that is similar to how anxious or depressed people tend to make negative judgments about ambiguous stimuli [87]. Whereas previously, as attachment theorists, we may have made sense of the relationship between behavioural flexibility, social stimuli and appraisal in terms of internal working models, we now suggest that epistemic trust is the mechanism via which humans’ behavioural flexibility arising from appraisals becomes compromised.

## Implications for prevention and intervention

Different approaches to BPD from a theoretical and practical point of view appear to be embarrassingly similar in terms of outcome [88, 89] in BPD. Based on the considerations outlined in this paper, we suggest that all effective treatments of BPD involve the same structure, namely that the re-emergence of epistemic trust requires three initially sequentially implemented but, as treatments unfold, increasingly concurrent forms of communication.

### Communication system 1

This entails the communication of therapeutic model-based content that indicates to the patient that the therapist has considerable knowledge as well as personal characteristics that may be highly valued by the patient. The knowledge communicated will naturally vary according to the treatment model (e.g. Transference Focused Psychotherapy will communicate information about primarily subtle intrapsychic relationships, while Dialectical Behavior Therapy will offer broader psychological constructs and coping strategies). Content analysis of all effective treatments reveal that the relationship of therapist and patient is supported by the former conveying a convincing understanding of the patient as an intentional agent which generates a sense of self-recognition. All evidence-based models of psychotherapy present models of mind, disorder and change that are accurate, helpful to patients and increase patients’ capacity for understanding. However, they also need to overcome the epistemic hypervigilance (‘not true’, ‘not relevant to me’) presented by the patient. So, besides the content, this stage involves a subtle and rich process of ostensive cueing. Thus, even at this relatively early stage the therapist must present their information with mentalizing in mind, establishing collaboration with the patient, demonstrating that they see the patient’s problems from their perspective, recognizing them as an agent, and with the attitude that the patient has things to teach the therapist. Through this, the therapist responds contingently to the patient. From the structural perspective we are presenting here, the therapist’s attempt to apply his/her model to interactions with the patient serves as an ostensive cue, which increases the patient’s epistemic trust and thus acts as a catalyst for therapeutic success. It does so to the extent that (a) the therapist is able to find and effectively transmit content that provides valuable ways for the patient to understand (mentalize) themselves and their reaction to others, and (b) the process of transmission involves the patient recognizing the truth and personal relevance of the content, so they become able to relax their epistemic mistrust.

### Communication system 2

Mentalizing may be a common factor in effective psychotherapies, but not in the sense that we originally intended [90]. It is not that, regardless of the therapeutic model, patients learn the ‘Esperanto’ of mentalizing, or even the altogether more appealing discourse of ‘plain old therapy’ [91]. The constant engagement of the patient by the therapist has several key features that are relevant to the restoration of epistemic trust. First, the therapist consistently recognizes the patient’s agentiveness, focuses on him/her as an actor and negotiates from the perspective of the patient’s self. Second, by marking the patient’s experiences, the therapist acknowledges the patient’s emotional state. Third, the therapist makes extensive use of ostensive cues to denote the personal relevance of the information transmitted and its generalizable social value. By mentalizing the patient effectively, the therapist models mentalizing, creating an open and trustworthy environment with low arousal. Structurally, a ‘virtuous cycle’ is put into motion: the therapist responds sensitively to the patient, the patient takes a step back from epistemic isolation, and the patient gradually begins to exercise his/her mentalizing skills, which, step by step, extend from the confines of the therapeutic context and generalize to his/her wider social context. This elicits an emotional reaction by the patient to the social context, giving the therapist further opportunity to respond sensitively. This process involves a complex and non-linear progression. Improving mentalizing is not its main goal, but the improved mentalizing that results from it enables the patient to start to approach and learn from their wider social context. Answering the question of why patients with a better capacity for mentalizing improve more in psychotherapy than those whose mentalizing is poorer helps us to understand the process. Mentalizing moderates the impact of therapeutic communications: a poorly mentalizing patient will frequently interpret the therapist’s ostensive cues erroneously, and epistemic trust is thus not established. With improved mentalizing, the therapist’s communications are appreciated and interpreted as trustworthy – and have the intended influence on the patient. The experience of having one’s subjectivity understood – of being mentalized – is a necessary trigger for being able to receive and learn from the social knowledge that has the potential to change one’s perception of oneself and the social world. The ‘gift’ of a mentalizing process in psychotherapy is to open up or restore the patient’s receptivity to broader social influence, which is a precondition for social learning and healthy development at any age.

### Communication system 3

The greatest benefit from a therapeutic relationship comes from the generalizing of epistemic trust *beyond* therapy, such that the patient can continue to learn and grow from other relationships. Social learning in the context of epistemic trust is (re)established, and this leads to salutogenesis. The third communication system is a process of opening the person’s mind via establishing epistemic trust (collaboration) so he/she can once again trust the social world by changing his/her expectations of it. This means that it is not just *what* is taught in therapy that helps the patient, but that the patient’s capacity for learning from social situations is rekindled. Enhanced mentalizing allows the patient to achieve improved social relationships and recognize who is a reliable and trustworthy source of information – that is, who one can ‘be friends with’. The improved epistemic trust and abandonment of rigidity enables learning from experience once again. So, therapeutic change is probably a consequence of how the patient comes to use their social environment, and not to what happens in therapy per se. The benefits of therapy remain contingent on what is accessible to patients in their particular social world. Therapeutic interventions are effective because they open the patient to social learning experiences which feed back in a virtuous cycle. If the environment is at least partly benign, therapy will ‘work’.

This third system – social learning in the context of epistemic trust – is, according to our thinking, the mechanism at work in the circular and self-perpetuating relationship between BPD and the social context. The conceptualization of the three communication systems outlined here involves an acknowledgment of the inherent limitations of clinical interventions in cases where the patient’s wider social environment does not support mentalizing. The implication of this is that what happens in any therapeutic intervention cannot on its own be expected to be enough to bring about any lasting significant improvement in the patient’s state. Indeed, in certain circumstances it would be maladaptive for the individual to develop epistemic trust and lower their social defences – for instance, in social environments characterized by high levels of aggression or violence, in which an external, non-reflective, rapidly responding affective focus on others as opposed to the self would be better prioritized as a survival strategy.

## Conclusions

Several features of the theoretical approach presented in this paper await further empirical confirmation, but according to the theory of epistemic trust and social learning, the lack of resilience, or positive appraisal, characteristic of individuals with BPD may be, in a sense, mislabelling. It may be more accurate to characterize BPD as an ‘emergency mode’ form of social understanding in which epistemic hypervigilance, distrust, or outright epistemic freezing is an adaptive consequence to the individual’s social environment. For various possible reasons, the individual has adopted negative appraisal mechanisms as a default. This is a highly socially oriented perspective on personal psychopathology. The key argument is that BPD (or other manifestations of the absence of psychological resilience) is the outcome of the ways in which the individual has learned to respond to the transmission of social knowledge within their own social environment.

Future research is needed to investigate these assumptions in more detail. This may also lead to the development of new prevention and intervention strategies, which are urgently needed, particularly given the increasing recognition of the need for prevention strategies for BPD [92, 93].

## Abbreviations

AMBIT: Adaptive mentalization-based integrative therapy
BPD: Borderline personality disorder
CAPSLE: Creating a Peaceful School Learning Environment
FACTS: Families and Carers Training and Support programme
PD: Personality disorder
